# Human Plasma Very Low-Density Lipoproteins Are Stabilized by Electrostatic Interactions and Destabilized by Acidic pH

**DOI:** 10.1155/2011/493720

**Published:** 2011-05-15

**Authors:** Madhumita Guha, Olga Gursky

**Affiliations:** Department of Physiology and Biophysics, School of Medicine, Boston University, Boston, MA 02118, USA

## Abstract

Very low-density lipoproteins (VLDL) are precursors of low-density lipoproteins (LDL, or “bad cholesterol”). Factors affecting structural integrity of VLDL are important for their metabolism. To assess the role of electrostatic interactions in VLDL stability, we determined how solvent ionic conditions affect the heat-induced VLDL remodeling. This remodeling involves VLDL fusion, rupture, and fission of apolipoprotein E-containing high-density lipoprotein-(HDL-) like particles similar to those formed during VLDL-to-LDL maturation. Circular dichroism and turbidity show that increasing sodium salt concentration in millimolar range reduces VLDL stability and its enthalpic component. Consequently, favorable electrostatic interactions stabilize VLDL. Reduction in pH from 7.4 to 6.0 reduces VLDL stability, with further destabilization detected at pH < 6, which probably results from titration of the N-terminal *α*-amino groups and free fatty acids. This destabilization is expected to facilitate endosomal degradation of VLDL, promote their coalescence into lipid droplets in atherosclerotic plaques, and affect their potential use as drug carriers.

## 1. Introduction

**Figure 1:**
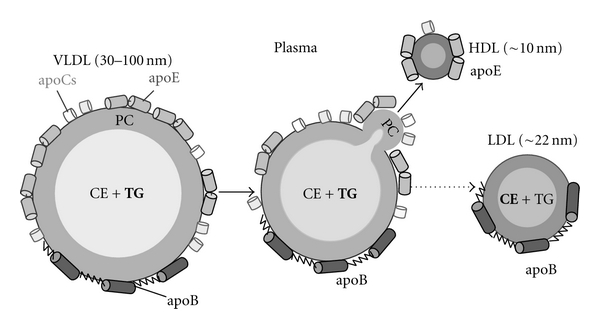
Cartoon showing metabolic remodeling of VLDL. Intact VLDL contain a core of apolar lipids, mostly triacylglycerides (TG) and some cholesterol esters (CE), surrounded by polar surface comprised mainly of phosphatidylcholines (PCs) and apolipoproteins. Exchangeable proteins, apoE (32 kD) and apoCs (6–9 kD), are comprised of amphipathic *α*-helical repeats and are shown in cylinders; the nonexchangeable apoB (550 kD) contains domains with predominantly *α*-helical or *β*-sheet structure. VLDL remodeling *in vivo *starts with TG hydrolysis by lipoprotein lipase; this produces excess surface that dissociates in the form of apoE-containing particles that join the pool of HDL [3, 4]. Similar particles are formed upon other VLDL perturbations such as heating [20]. Remodeling of VLDL eventually converts them to LDL; each LDL contains one copy of apoB as its major protein. Dissociation of apoE and some lipids from the apoB-containing particles also occurs during endosomal degradation of VLDL [10–12].

Very low-density lipoproteins (VLDL) are the major plasma carriers of fat (triacylglycerides, TG) and direct metabolic precursors of low-density lipoproteins (LDL, or “bad cholesterol”). Elevated plasma levels of TG are a hallmark of the metabolic syndrome and a risk factor for atherosclerosis [1–6]. Human plasma VLDL are heterogeneous particles (*d*~35–100 nm) that contain a large apolar core comprised mainly of TG and cholesterol esters and polar surface comprised of apolipoproteins (apos) and a phospholipid monolayer (Figure 1). Each VLDL particle contains one copy of the nonexchangeable (water-insoluble) apoB (550 kDa) that comprises nearly one half of its total protein content [5]; the other half is comprised of multiple copies of the exchangeable proteins, apoE (32 kDa) and apoCs (6–9 kD). During VLDL metabolism (recently reviewed in [6]), core TG are enzymatically hydrolyzed to produce free fatty acids that are used as an energy source by various tissues. Upon TG hydrolysis, the lipoprotein core shrinks and the excess surface material fissions in the form of small apoE-containing particles that join the plasma pool of high-density lipoproteins (HDL, or “good cholesterol”, *d*~10 nm) [7–9] (Figure 1). Eventually, VLDL are converted to LDL (*d*~22 nm) that contain one copy of apoB comprising >90% of their total protein content. 

Similar to LDL, VLDL uptake by the cells involves receptor-mediated whole-particle endocytosis. In contrast to LDL that are degraded in the lysosomes, VLDL degradation is a complex process that starts in early endosomes with dissociation of apoE together with a fraction of phospholipids; apoE eventually gets recycled, while the remaining apoB-containing particle undergoes lysosomal degradation [10–12]. Such receptor-mediated uptake of the apoB-containing lipoproteins downregulates cell cholesterol biosynthesis and is antiatherogenic. Alternatively, LDL and VLDL can bind to the arterial wall proteoglycans where they get retained and modified by hydrolytic and oxidative enzymes in the subendothelial space [13–15]. Such retention of the apoB-containing lipoproteins is believed to be the key initiating step in atherogenesis ([16] and references therein). It leads to a cascade of proatherogenic responses, including lipoprotein fusion into lipid droplets that are found in atherosclerotic plaques [17–19]. Such droplets are readily taken up by macrophages, which promotes formation of foam cells and progression of atherosclerosis ([19] and references therein). Our goal is to provide the energetic and structural basis for understanding key determinants for structural integrity of VLDL assembly and its remodeling into large and small particles and lipid droplets. 

Because of the experimental difficulties in analyzing structural stability of large heterogeneous lipid-loaded particles, studies of VLDL stability have been limited to our own work performed in 10 mM Na phosphate, pH 7.6 [20]. The results showed that thermal denaturation of VLDL is a complex kinetically controlled transition with two kinetic phases reflecting distinct morphologic transformations. The first phase involves VLDL fusion and dissociation of small spherical apoE-containing particles (*d* = 7–15 nm) whose size, density, and biochemical composition closely resemble a subclass of plasma HDL formed during metabolic remodeling of VLDL (Figure 1). The second phase involves complete lipoprotein disintegration and coalescence into lipid droplets whose size and morphology resemble lipid droplets found in atherosclerotioc plaques [17]. Hence, thermal denaturation of VLDL mimics key aspects of their metabolic remodeling *in vivo* and provides a useful model for elucidating the energetic and structural basis for this remodeling [20]. 

As neutral lipids comprise over 80% of the total VLDL mass, hydrophobic interactions are likely to dominate VLDL stability. However, electrostatic interactions are also likely to contribute, as suggested by the presence of small amounts of anionic lipids in VLDL, mainly free fatty acids and phosphatidylinositol, as well as by the sequence properties of the class-A amphipathic *α*-helices that form the major lipid surface-binding motif in the exchangeable apolipoproteins and are also found in the nonexchangeable apoB on VLDL [21]. Characteristic radial distribution and high content of charged residues in class-A helices (30–50% as compared to ~15% in typical globular proteins) facilitate formation of multiple salt bridges. These and other electrostatic interactions, which are amplified by the low dielectric at the lipid surface (**ε**~10 as compared to 80 in water), have been predicted [22] and observed to significantly affect the stability of model and plasma HDL [23–25]. Furthermore, charge-charge interactions are central to many functional interactions of lipoproteins with their metabolic partners, including VLDL interactions with lipophilic plasma enzymes, cell receptors, and arterial wall proteoglycans ([26–29] and references therein). Here, we explore the role of electrostatic interactions in structural stability and remodeling of human VLDL.

This paper reports the effects of solvent ionic conditions (pH 5.7–8.2, 1–150 mM Na salt) on thermal denaturation of human VLDL. The results reveal that (i) electrostatics interactions provide a large favorable enthalpic contribution to VLDL stability and (ii) reduction in pH from mildly basic to mildly acidic destabilizes VLDL and accelerates their remodeling. We propose a plausible explanation for these effects and postulate that reduction in VLDL stability upon reduction in pH, which occurs upon receptor-mediated VLDL transfer from plasma (pH 7.4) to early endosomes (pH~6) [10–12] or upon VLDL retention in the arterial wall and macrophage-induced acidification ([30] and references therein), may have important implications for VLDL metabolism.

## 2. Materials and Methods

### 2.1. VLDL Isolation and Characterization

**Figure 2:**
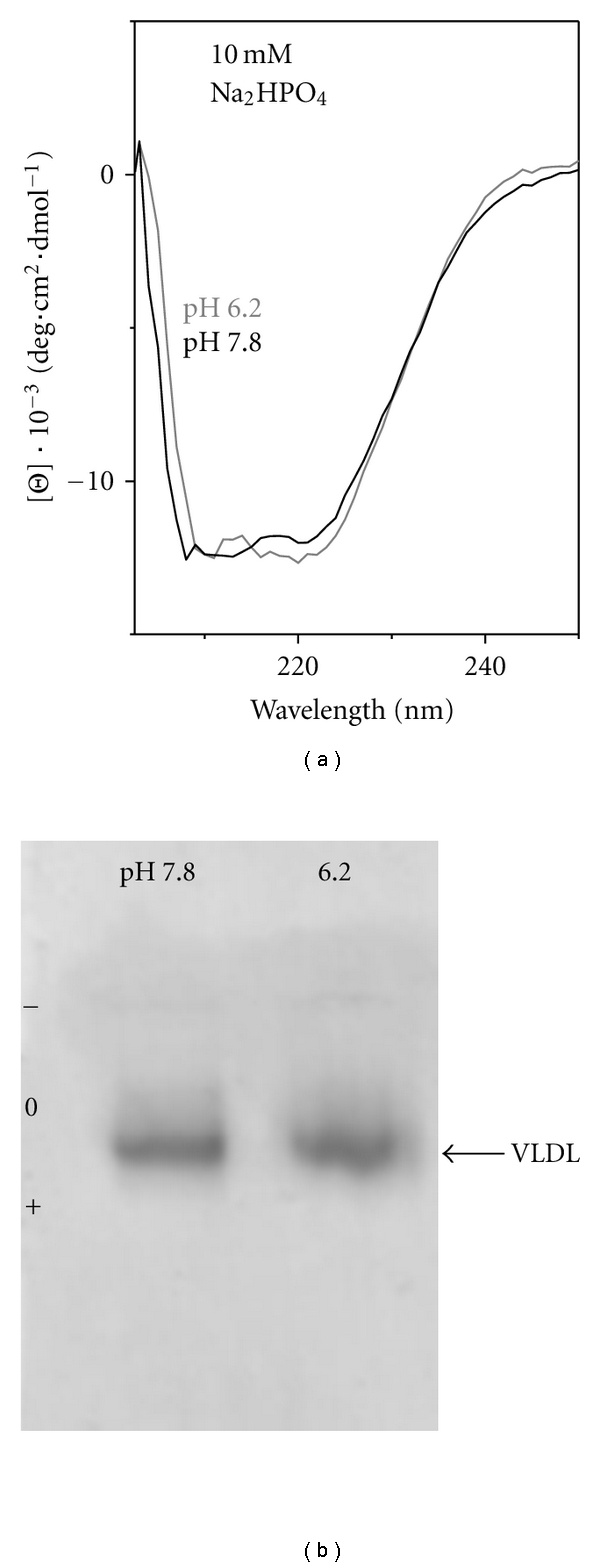
Change from mildly basic to mildly acidic conditions does not affect the protein conformation or the net change on VLDL. VLDL solutions in 10 mM Na phosphate buffer at pH 6.2 or 7.8 were analyzed by far-UV CD spectroscopy for the apolipoprotein secondary structure (a) or by agarose gel electrophoresis for the net charge on the lipoprotein (b).

Single-donor human VLDL were isolated as described [20] from EDTA-treated plasma of six healthy volunteer donors according to regulations of the Institutional Review Board. Briefly, VLDL were isolated by density gradient centrifugation in the density range 0.94–1.006 g/mL [31]. Total VLDL migrated as a single band on the agarose gel (Figure 2). Total human VLDL are comprised of two main subclasses, VLDL_1_ (*d* = 60–100 nm) and VLDL_2_ (*d* = 35–60 nm), containing one molecule of apoB per particle and numerous copies of apoE and apoCs (greater in larger particles). To improve sample homogeneity and to minimize light scattering in spectroscopic experiments, smaller VLDL_2_  particles were isolated by an additional round of centrifugation at 40,000 rpm for 30 min as described [20] and were used for further studies. The VLDL solution containing about 2 mg/mL protein was dialyzed against standard buffer (10 mM Na phosphate, 0.25 mM EDTA, 0.02% NaN_3_, pH 7.6). This stock solution was stored in the dark at 4°C and was used in 2-3 weeks during which we detected no change in VLDL charge by agarose gel, no protein degradation by SDS PAGE, and no changes in the protein conformation or particle stability by CD spectroscopy. Even though plasma lipoproteins isolated from different batches showed small batch-to-batch variations in stability reflecting small donor-specific variations in lipoprotein composition, the overall tends reported in this paper were similar for all VLDL batches explored. Therefore, lipoprotein heterogeneity did not affect the key conclusions of this study.

VLDL subjected to various thermal treatments were visualized at 25°C by negative staining electron microscopy (EM) using a CM12 transmission electron microscope (Philips Electron Optics) as described [20].

### 2.2. Solvent Conditions

Solvent ionic conditions explored in our thermal denaturation studies ranged from 1 to 150 mM Na salt, pH 5.7–8.2. At mildly acidic pH in ≥100 mM salt, VLDL were destabilized to such an extent that their decomposition and lipid phase separation occurred at ambient temperatures; this limited our experimental analysis of the pH effects to low-salt conditions. Similarly, at pH < 6.0 VLDL destabilization was observed at ambient temperatures in any salt concentration (including 150 mM NaCl), preventing quantitative studies of VLDL stability at these low pH. Hence, the results reported here are limited to pH ≥ 6.0. For pH studies, VLDL stock solution was dialyzed against 10 mM Na phosphate buffer varying in pH from 6.0 to 8.2. For studies of the ionic strength effects, buffered solution of NaCl, Na_2_SO_4_ or Na phosphate at pH 7.6 was added to VLDL stock solution to a final salt concentration ranging from 1 to 150 mM. 

To test the effects of salts other than Na phosphate and to avoid the effects of buffer saline, we attempted to use dilute TRIS buffer at pH 7.7. The melting data progressively shifted to lower temperatures upon increasing TRIS concentrations in low millimolar range, indicating VLDL destabilization (see Figure S1 in Supplementary Materials available online at doi: 10.1155/2011/493720). Addition of salt caused further destabilization (Figure S1), leading to rapid lipid phase separation upon heating which precluded accurate analysis of the VLDL stability. Similar destabilizing effect of TRIS at low mM concentrations was observed in LDL, HDL, and in apoA-I isolated from HDL (unpublished data), suggesting strongly that TRIS interacts unfavorably with this and other apolipoproteins. Therefore, in our structural stability studies we used 10 mM Na phosphate buffer rather than TRIS.

### 2.3. Circular Dichroism (CD) and Turbidity

CD and turbidity data were recorded using an AVIV spectropolarimeter with thermoelectric temperature control as described [20]. Briefly, the CD data were recorded using VLDL solutions of about 0.1 to 0.15 mg/mL protein concentrations in far-UV (190–250 nm) or about 0.5 mg/mL protein in near-UV/vis (250–500 nm). Since earlier we showed that changes in VLDL concentration significantly affect the amplitude of the heat-induced structural transition but not its apparent melting temperature *T_m_* [20] (which implies that particle collision does not provide a rate-limiting step in VLDL remodeling), the key conclusions of our study do not depend on the VLDL concentrations used. Far-UV CD spectra were normalized to protein concentration (based on the total protein concentration in mg/mL and assuming an average molecular weight per residue of 113 Da) and expressed as molar residue ellipticity, [Θ]. Quantitative secondary structural analysis was not carried out because of distortions in the CD spectra of large particles. Heat-induced changes in turbidity were monitored by dynode voltage *V* in CD experiments as described [20, 32], either at 220 nm or at 230 nm, together with the CD data recorded at the same wavelength (220 nm for secondary structure unfolding and 320 nm for repacking of apolar lipids, described below). Turbidity data recorded at either wavelength were qualitatively similar. 

In the melting experiments, CD and turbidity data, Θ(*T*) and *V*(*T*), were recorded simultaneously at 220 or 320 nm during sample heating and cooling from 25 to 98°C at a constant rate of 11°C/h. The apparent transition temperatures *T_m_* were determined from the peak positions in the first derivative of the heating data,* dV*(*T*)/*dT *or *d*Θ(*T*)/*dT*; the accuracy of this determination was 1°C. Turbidity was used to monitor heat-induced increase in the particle size upon VLDL fusion and rupture, and CD was used to monitor VLDL rupture and release of apolar core lipids that coalesce into large lipid droplets. Earlier we showed that such droplet formation, which was detected upon rupture of all core-containing human lipoproteins, induces a large negative CD peak centered at 320 nm [33]. The amplitude of this peak increased with increasing the lipoprotein size, HDL < LDL < VLDL, and the size of the resulting droplets. This induced CD apparently results from the apolar lipids such as TG, cholesterol esters, and carotenoids, upon their escape from the lipoprotein core and repacking in lipid droplets. In VLDL, this negative CD peak was so large that it extended to far-UV and dominated the heat-induced changes in Θ_220_(*T*) [20]. Therefore, in contrast to conventional use of far-UV CD for monitoring protein unfolding, we use Θ_220_(*T*) to monitor lipid repacking upon VLDL rupture and formation of lipid droplets [20].

In kinetic temperature-jump (*T*-jump) experiments, VLDL denaturation was triggered at *t* = 0 by rapid heating from 25°C to a higher constant temperature (70–95°C). The time course of VLDL fusion and rupture was monitored by turbidity (dynode voltage), *V*(*t*). Data analysis was carried out by using an Arrhenius model. Briefly, the kinetic data recorded at each temperature were approximated with a multiexponential decay function:

(1) $$V\left(t\right)=\;A_{1}\cdot \exp\;\left({-k}_{1}t\right)+\;A_{2}\cdot \exp\;\left({-k}_{2}t\right)+\cdots .$$

Here, *A_i_* is the amplitude, and *k_i_* is the temperature-dependent rate constant of the *i*th kinetic phase. Since VLDL denaturation is irreversible, the reaction rate equals the denaturation rate. The rate constants *k_i_*(*T*) were determined by fitting the *V*(*t*) data with the multi-exponentials, and the Arrhenius activation energy (enthalpy) *E*_*a*_ for each kinetic phase was determined from the slope of the Arrhenius plot, *RT* ln  *k*_*i*_(*T*)* versus* 1/*T*. Changes in the Gibbs free energy of stability, *δ*ΔG* = *RT* *δ*[ln  *k*(*T*)], were assessed from the shifts in these plots. All experiments in this work were repeated 3–6 times to ensure reproducibility.

## 3. Results

### 3.1. Effects of pH

**Figure 3:**
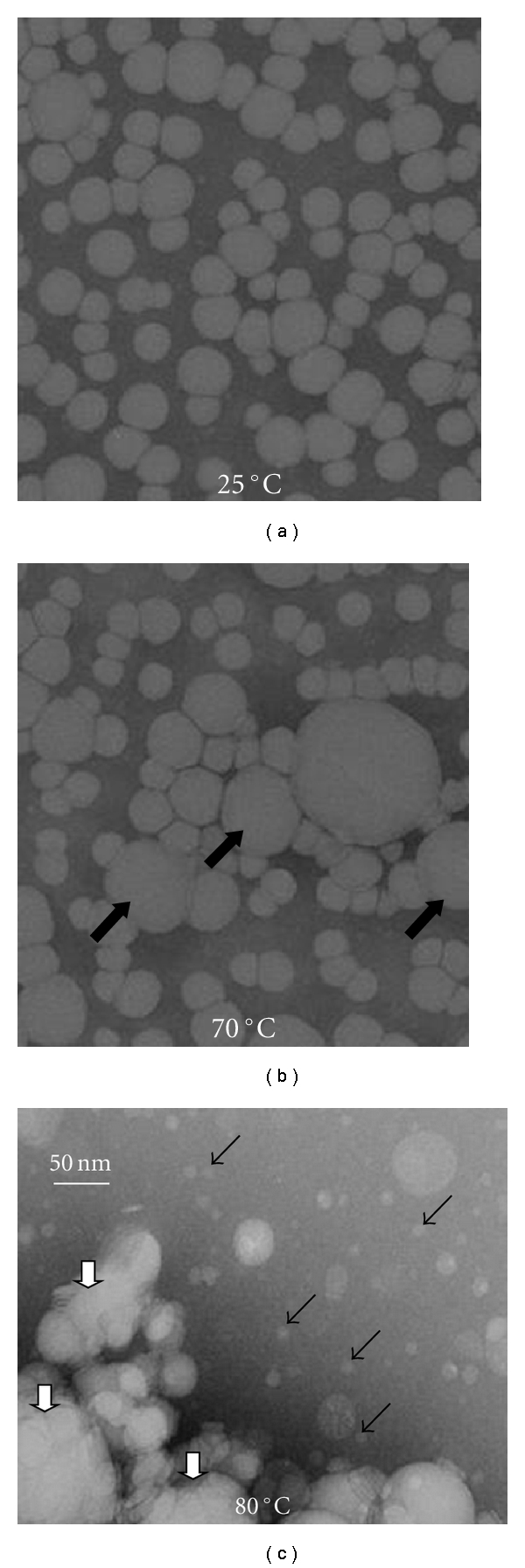
Negative stain electron microscopy of intact and heated human plasma VLDL. Buffer conditions are 10 mM Na phosphate, pH 6.2. Lipoproteins were intact (a) or heated at a constant rate of 11°C/h to 70°C (b) or 80°C (c). Large black arrows indicate fused VLDL, large white arrows indicate lipid droplets formed upon VLDL rupture (such rupture is accompanied by loss of lipoprotein morphology and repacking of apolar core lipids indicated by near-UV CD), and small arrows point to small HDL-size particles whose detailed biophysical and biochemical analysis was reported earlier [20].

**Figure 4:**
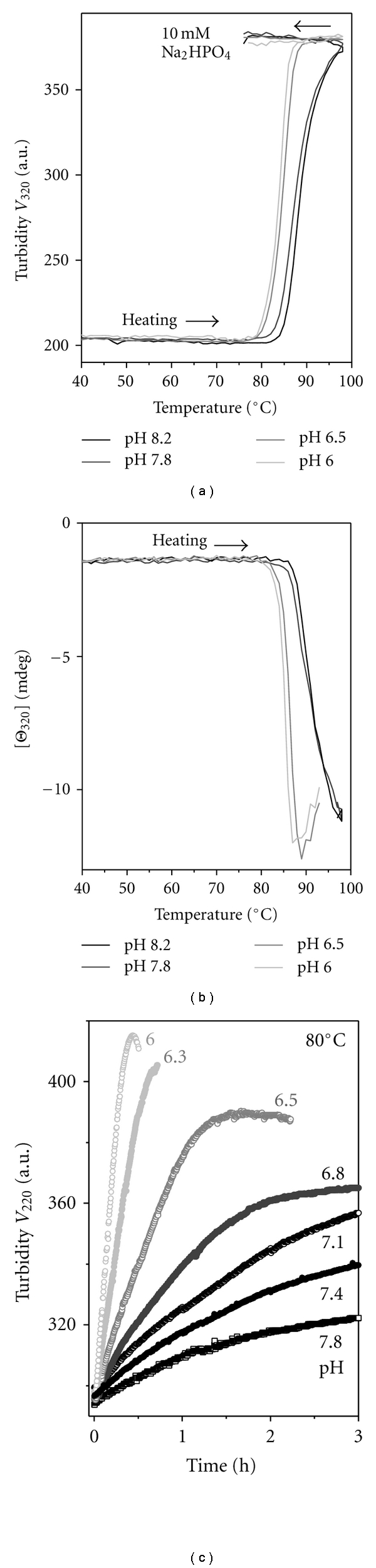
Effects of pH on VLDL stability. Thermal denaturation of VLDL in 10 mM Na phosphate buffer at pH 8.2–6.0 (selected pH values are shown) was analyzed in the melting ((a) and (b)) and kinetic CD experiments (c). In the melting experiments, VLDL solutions were heated at a rate of 11°C/h. Changes in turbidity (a) and circular dichroism (b) were monitored at 320 nm for increase in the particle size upon lipoprotein fusion and coalescence into droplets (a) and lipoprotein rupture and repacking of apolar lipids in droplets (b). In temperature-jump experiments (c), VLDL heat denaturation was triggered at time *t* = 0 by a rapid increase in temperature from 25 to 80°C, and the denaturation time course was monitored by turbidity at 220 nm.

VLDL structure and stability were analyzed in 10 mM Na phosphate buffer varying in pH from 6.0 to 8.2. The results showed that such variations produced no detectable changes in the secondary structural content of VLDL proteins assessed by far-UV CD (Figure 2(a)), in the aromatic packing in these proteins assessed by near-UV CD (data not shown), or in the net charge on VLDL assessed by agarose gel electrophoresis (Figure 2(b)). Also, the size and morphology of intact VLDL remained invariant in this pH range, as evident from the electron micrographs recorded of VLDL under acidic (Figure 3(a)) or basic conditions and ambient temperatures [20]. Furthermore, EM data showed that the heat-induced remodeling of VLDL proceeds via similar stages at various solvent ionic conditions explored, such as pH 6.2 and pH 7.6 shown in Figure 3 and in [20], respectively. This remodeling involves VLDL fusion, rupture and fission of small apoE-containing HDL-like particles described before [20]. The results of the current work revealed that the apparent temperatures of these structural transitions are pH dependent. To test this pH dependence, VLDL samples (0.1 mg/mL protein in 10 mM Na phosphate buffer at pH 6.0–8.2) were heated at a constant rate of 11°C/h, and CD and turbidity were measured simultaneously at 320 nm to monitor VLDL rupture and lipid repacking upon coalescence into droplets (by CD) and increase in the particle size due to fusion and lipid droplet formation (by turbidity). The results revealed that the reduction in pH from pH 8.2 to 6.0 led to a low-temperature shift in the CD and turbidity heating data by more than −10°C (Figures 4(a) and 4(b)), indicating a large reduction in VLDL stability at acidic pH.

This notion was further tested in kinetic *T*-jump experiments. VLDL denaturation was triggered by a rapid heating from 25°C to higher constant temperatures, and its time course was monitored by turbidity at 220 nm, *V *_220_(*t*).  Figure 4(c) illustrates a subset of data recorded at pH 6.0–7.8 in *T*-jumps to 80°C. These and other kinetic data recorded in the range of pH 6.0–8.2 clearly show that reduction in pH progressively accelerates VLDL denaturation and, hence, is destabilizing. 

Earlier, we carried out Arrhenius analysis of the *T*-jump data recorded of VLDL 10 mM Na phosphate at pH 7.6 and determined the activation energy (enthalpy) of denaturation under these conditions, *E*_*a*_ = 53 ± 7 kcal/mol [20]. Similar analysis at acidic pH was hampered by VLDL destabilization that led to rapid lipoprotein rupture and lipid phase separation, which manifested itself as a loss of spectroscopic signal at advanced stages of heat denaturation (grey lines in Figures 4(b) and 4(c)). This precluded accurate turbidity measurements necessary to determine the denaturation rate constants *k*(*T*) and thereby hampered quantitative analysis of VLDL stability at acidic pH. As a ballpark estimate of the pH-induced reduction in VLDL stability, we compared the *T*-jump data recorded at 80°C in 10 mM Na phosphate at pH 7.4 or pH 6.0. VLDL denaturation proceeds at least 20 times faster at pH 6.0 as compared to pH 7.4, *k*_pH 7.4_/*k*_pH 6.0_  ≥ 20. Therefore, the reduction in pH from 7.4 to 6.0 corresponds to a reduction in kinetic stability ΔG* by *δ*ΔG* = −*RT* · *δ*(ln  *k*) = −*RT* · ln  [*k*_pH 7.4_/*k*_pH 6.0_]≥−1.8 kcal/moL.

Qualitatively, reduction in VLDL stability at acidic pH was confirmed by negative staining EM. Electron micrographs consistently showed that the heat-induced remodeling of VLDL proceeded via similar stages at different pH but occurred faster at acidic as compared to basic conditions. For example, at pH 6.2, VLDL heating at a rate of 11°C/h to 70°C led to substantial particle fusion (Figure 3(b)), yet similar heating at pH 7.6 caused no morphologic changes in VLDL. Further heating to 80°C led to formation of large lipid droplets and small HDL-size particles at pH 6.2 (Figure 3(c)), yet at pH 7.6 only fused particles were observed (similar to those in Figure 3(b)). Taken together, our EM, CD, and turbidity data recorded in the melting and kinetic experiments clearly showed that reduction in pH from mildly basic to mildly acidic accelerates heat-induced VLDL remodeling and, hence, destabilizes VLDL.

### 3.2. Effects of Salt

Effects of ionic strength on the structural stability of VLDL were first determined in dilute Na phosphate buffer at pH 7.6. At this pH and room temperature, changes in the salt concentration from 1 to 150 mM Na_2_HPO_4_ or from 0 to 100 mM NaCl or Na_2_SO_4_ produced no changes in VLDL morphology or in the protein conformation that could be detected by EM or by far- and near-UV CD, respectively. Furthermore, EM data showed that the products of the heat-induced structural transitions, including VLDL fusion, rupture and fission of small HDL-size particles, are similar under any solvent ionic conditions explored. However, CD and turbidity data recorded in the melting and kinetic experiments revealed large effects of salt on the temperature range and the rate of VLDL denaturation.

**Figure 5:**
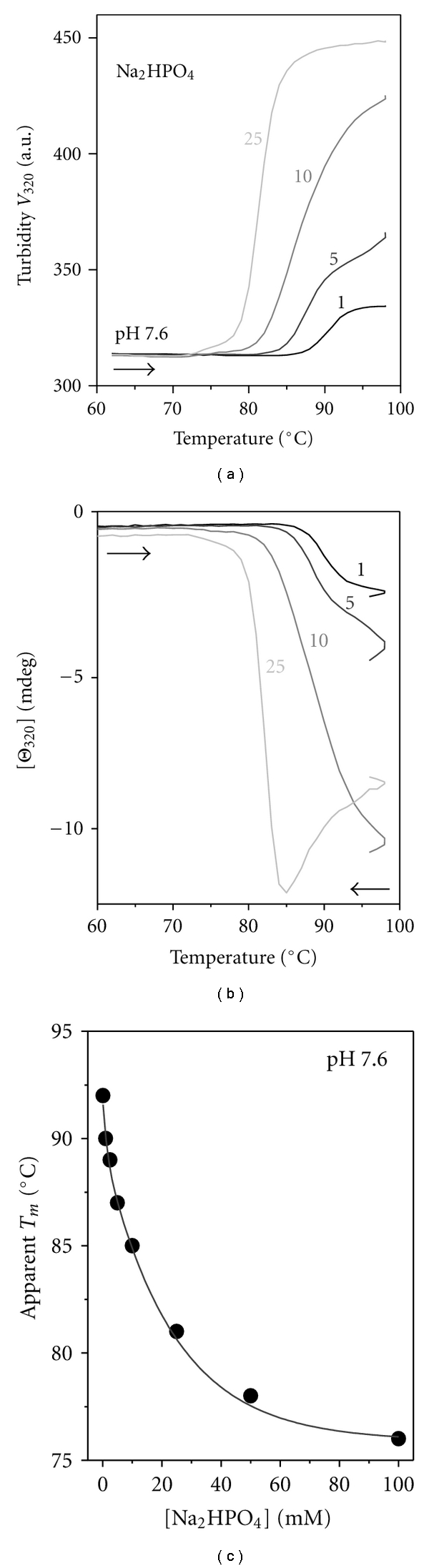
Effects of millimolar concentrations of Na phosphate on the structural stability of VLDL assessed in the melting experiments. VLDL solutions containing 1–25 mM Na phosphate (as indicated) at pH 7.6 were heated at a constant rate of 11°C/h. The turbidity (dynode voltage) and CD melting data, *V *_320_(*T*) and Θ_320_(*T*), were recorded simultaneously at 320 nm. (a) Turbidity was used to monitor increase in the particle size due to fusion and coalescence into lipid droplets, and (b) CD was used to monitor repacking of apolar core lipids upon VLDL rupture and coalescence into lipid droplets [20, 33]. (c) The apparent melting temperature *T_m_*, which was determined from the peak position in the 1st derivative of the turbidity melting data such as those in (a), is plotted as a function of Na phosphate concentration. Data fitting with a monoexponential decay function (solid line, *K* = 0.05 mM^−1^) is characteristic of the ionic screening effect [34], with *K* providing a measure of macromolecular sensitivity to ionic screening. A similar effect of salt was observed in human HDL [24].

First, the melting data were recorded from VLDL solutions varying in Na phosphate concentration from 1 to 150 mM under otherwise identical conditions (0.1 mg/mL protein, pH 7.6). The samples were heated from 25 to 98°C at a rate of 11°C/h and CD, and turbidity melting data, Θ_320_(*T*) and V_320_(*T*), were recorded simultaneously at 320 nm. Selected data are presented in Figures 5(a) and 5(b). These and other data clearly show that increasing Na phosphate concentration from 1 to 25 mM progressively shifts VLDL fusion and rupture to lower temperatures by up to −10°C, indicating salt-induced reduction in stability. These low-temperature shifts were accompanied by a progressive increase in the amplitude and the apparent cooperativity of the transition (Figures 5(a) and 5(b)), suggesting formation of larger lipid droplets in higher-salt solutions; such large droplets were detected by electron microscopy at ≥25 mM Na phosphate (data not shown). Moreover, heat-induced VLDL rupture in solutions at ≥25 mM Na phosphate led to a rapid lipid phase separation that was visible by eye. This manifested itself as a signal loss, for example, reduction in CD amplitude following VLDL rupture at high temperatures (light grey line in Figure 5(b)), precluding accurate spectroscopic measurements in high-salt solutions at advanced stages of VLDL denaturation.

The apparent temperature *T_m_*  of the heat denaturation was determined from the peak position in the 1st derivative of the turbidity data, *dV*(*T*)*/dT*. These data were recorded in 1–100 mM Na phosphate at pH 7.6.  Figure 5(c) shows *T_m_* plotted as function of salt concentration. This plot illustrates a large reduction in *T_m_* from 92 to 76°C upon increasing Na phosphate concentration from 1 to 100 mM. The dominant effect of salt at these concentrations is ionic screening. Furthermore, the plot of *T_m_* versus salt concentration was well approximated by an exponential decay function (solid line in Figure 5(c)), which is characteristic of ionic screening by diffuse counterions [34, 35]. Thus, the results in Figure 5(c) suggest strongly that the salt-induced reduction in stability results from ionic screening of favorable electrostatic interactions on VLDL surface.

Next, we tested whether the salt-induced decrease in the water activity contributes to reduction in VLDL stability. The hydrophobic effect of salt on macromolecular stability usually becomes significant above 0.1 M salt [34, 35] and can be mimicked by nonionic compounds such as sugars. To test this effect, CD and turbidity melting data were recorded from VLDL solutions containing 0.1 mg/mL protein, 10 mM Na phosphate buffer at pH 7.6, and 0 or 10% sucrose. Such sucrose solution has the water activity similar to that of the physiological saline (~150 mM NaCl). The melting data recorded of VLDL in 0 and 10% sucrose under otherwise identical conditions fully overlapped (not shown). Therefore, at and below physiologic salt concentrations, salt-induced changes in the water activity caused no detectable changes in VLDL stability. Taken together, our results indicate that the reduction in VLDL stability upon increasing salt concentration results from ionic screening of favorable electrostatic interactions. 

**Figure 6:**
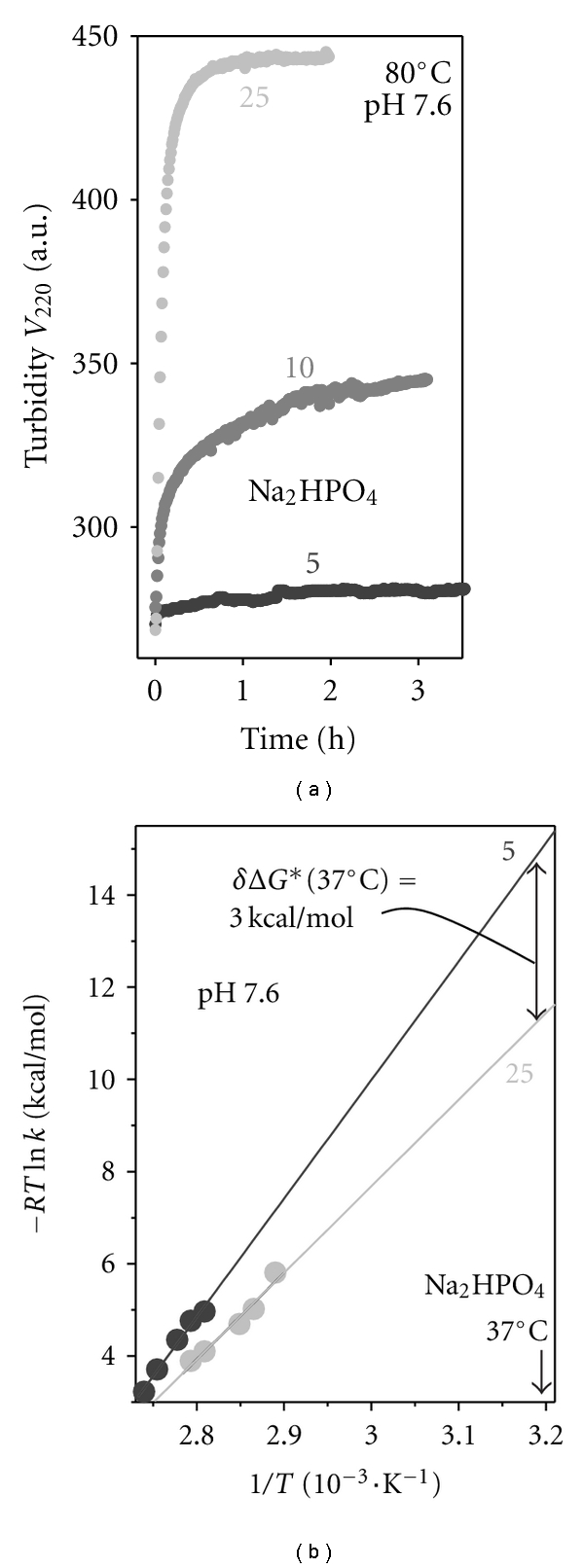
Kinetic analysis of the effects of Na phosphate on VLDL stability. Thermal denaturation of VLDL (0.1 mg/mL protein, 5–25 mM Na phosphate as indicated, pH 7.6) was triggered in temperature-jumps from 25°C to larger constant values of 70–95°C. The time course of denaturation was monitored by turbidity at 220 nm, *V *_220_(*t*). (a) Representative data recorded in *T*-jumps to 85°C; Na phosphate concentrations are indicated. (b) Arrhenius analysis of the *T*-jump data recorded in 5 mM or in 25 mM Na phosphate. Solid lines show data fitting by linear functions; the slopes of these functions correspond to the activation energy (enthalpy)  *E*_*a*_ of VLDL denaturation. Linear extrapolation of the Arrhenius plots to 37°C suggests that VLDL stability decreases by about 3 kcal/mol upon increasing Na phosphate concentration from 5 to 25 mM (double arrow).

The effect of salt on VLDL stability was further explored in kinetic experiments. VLDL samples (0.1 mg/mL protein, pH 7.6) containing 5–25 mM Na phosphate were subjected to *T*-jumps from 25°C to 70–98°C. VLDL fusion and rupture were monitored by turbidity at 220 nm, *V *_220 _(*t*).  Figure 6(a) illustrates selected *T*-jump data recorded at 85°C. Comparison of such data recorded at this and other temperatures clearly shows that addition of salt accelerates VLDL fusion and rupture and, hence, reduces VLDL stability. To quantify this effect, we used Arrhenius analysis. The *V *_220 _(*t*) data recorded in 5 to 25 mM Na_2_HPO_4_  were approximated by exponential decay functions, the unfolding rates *k*(*T*) were determined, and the Arrhenius plots, −*RT*ln *k*(*T*) *versus 1/T*, were obtained. Monoexponential denaturation kinetic was observed at ≥20 mM salt, while at 2–10 mM salt two exponents were required to fit the *V *_220 _(*t*) data, suggesting two distinct kinetic phases in VLDL denaturation. A similar two-phase kinetics was observed in our earlier study of VLDL stability in 10 mM Na phosphate, pH 7.6 [20]. In that study, CD, turbidity, and EM data showed that the 1st phase involves fusion of intact VLDL and fission of HDL-like particles and the 2nd phase involves lipoprotein rupture and release of apolar core lipids [20]; the Arrhenius plots for these two phases were parallel with a slope corresponding to the activation energy (enthalpy) *E*_*a*_ = 53 ± 7 kcal/mol. In the current study, we used the 1st kinetic phase (i.e., fusion of intact VLDL and fission of HDL-size particles) for comparison of the effect of salt on VLDL stability. The results showed that increasing Na phosphate concentration from 5 to 25 mM leads to a large decrease in the slope of the Arrhenius plot which corresponds to a decrease in the activation energy of VLDL denaturation, from *E*_*a*_ = 75 ± 15 kcal/mol in 5 mM Na_2_HPO_4_ to *E*_*a*_ = 48 ± 7 kcal/mol in 25 mM Na_2_HPO_4_ (Figure 5(c)). Linear extrapolation of the Arrhenius plots to 37°C suggests that increasing Na phosphate concentration from 5 to 25 mM leads to a reduction in kinetic stability by about *δ*ΔG* (37°C) = −3 ± 0.5 kcal/mol (Figure 6(b), double arrow). In summary, our CD, turbidity, and EM data recorded in the melting and kinetic experiments consistently show a large reduction in VLDL stability upon increasing Na phosphate concentration. The Arrhenius analysis demonstrates that this reduction in stability is enthalpy driven (Figure 6(b)). The latter is consistent with the enthalpy-driven ionic screening effect of salt (which is indicated by melting data in Figure 5(c)), as opposed to the entropy-driven salt effect on the water activity.

**Figure 7:**
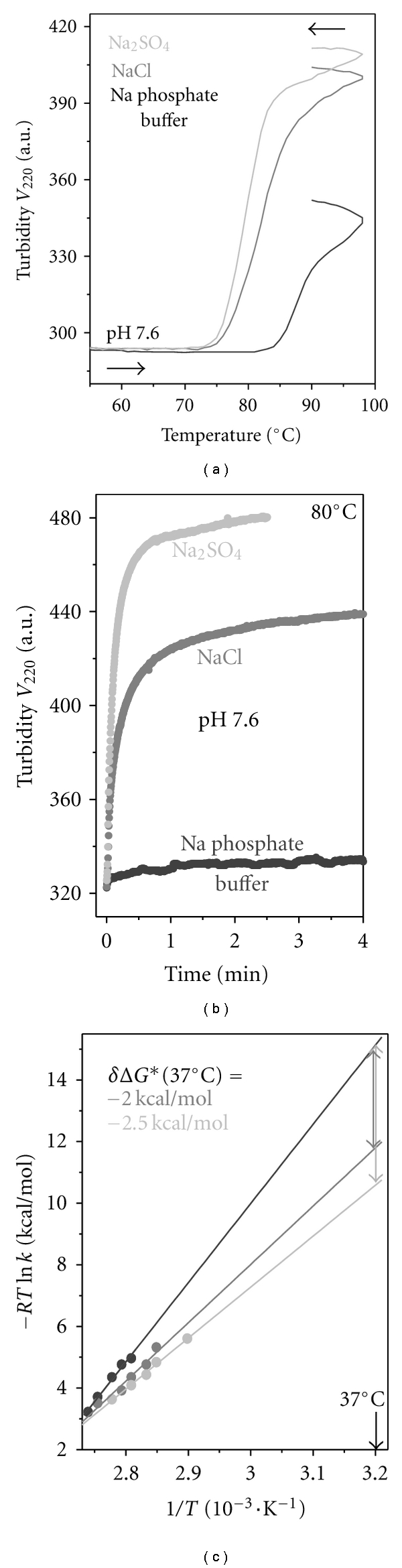
Effects of Na salts on thermal stability of VLDL. VLDL solutions (0.1 mg/mL protein concentration, 5 mM Na phosphate buffer, pH 7.6) containing buffer alone (black) or together with 10 mM NaCl (grey) or Na_2_SO_4_ (light grey) were subjected to melting (a) or kinetic experiments ((b) and (c)). The melting data were recorded by turbidity during sample heating at a constant rate of 11°C/h (a). The kinetic data were recorded in *T*-jumps from 25°C to higher temperatures, such as 85°C (b). Arrhenius analysis of the *T*-jump data shows that, similar to Na phosphate (Figure 6(b)), increasing the NaCl or Na_2_SO_4_ concentration leads to a decrease in the slope of the Arrhenius plot, indicating a decrease in the activation energy *E*_*a*_. Linear extrapolation of the Arrhenius plots suggests that the kinetic stability of VLDL at 37°C decreases by about 2 kcal/mol upon addition of 10 mM NaCl and by about 2.5 kcal/moL upon addition of 10 mM Na_2_SO_4_ (double arrows).

To assess the effects of other Na salts on thermal denaturation of VLDL, we used 10 mM NaCl or Na_2_SO_4_  in 5 mM Na phosphate buffer at pH 7.6. TRIS buffer could not be used in these studies because of its unfavorable interactions with lipoproteins and apolipoproteins (see Section 2 and Figure S1). Turbidity melting data recorded of VLDL at a heating rate of 11°C/h showed a large low-temperature shift by about −15°C in the presence of 10 mM NaCl and an even larger shift in 10 mM Na_2_SO_4_, indicating reduction in VLDL stability by these salts (Figure 7(a)). Furthermore, kinetic data recorded by turbidity in *T*-jumps to 85°C (Figure 7(b)) or other temperatures showed faster VLDL denaturation in 10 mM NaCl and, particularly, Na_2_SO_4_ as compared to buffer alone. Quantitative analyses of the *T*-jump data recorded in 0–10 mM NaCl or Na_2_SO_4_ were performed, and the Arrhenius plots were extrapolated to near-physiologic temperatures (solid lines in Figure 7(c)). The results showed that, at 37°C, VLDL stability changes by about *δ*ΔG∗ (37°C) = −2 kcal/mol in the presence of 10 mM NaCl and by about −2.5 kcal/moL in 10 mM Na_2_SO_4_ (double arrows in Figure 7(c)). A large enthalpic contribution to this reduction in stability is evident from the large differences in the slopes of the Arrhenius plots (Figure 7(c), black, gray, and light-gray lines), which correspond to activation energies *E*_*a*_ = 75 ± 12 kcal/mol in 5 mM Na phosphate buffer alone, *E*_*a*_ = 60 ± 7 kcal/mol in buffered 10 mM NaCl solution, and *E*_*a*_ = 50 ± 7 kcal/mol in buffered 10 mM Na_2_SO_4_ solution. These effects are comparable to the destabilizing effect of Na phosphate on VLDL (Figure 6). In summary, different Na salts have similar but not identical destabilizing effects on VLDL, suggesting the role of the anions in the electrostatic screening. These effects are enthalpy driven and result from ionic screening of favorable electrostatic interactions on VLDL surface.

## 4. Discussion

### 4.1. Favorable Electrostatic Interactions Stabilize VLDL: Comparison with LDL and HDL

The results of our CD, turbidity, and EM studies revealed that increase in Na salt concentration from 10 to 100 mM and/or reduction in pH from 8.2 to 6.0 destabilize VLDL without changing the nature of their thermal remodeling (Figures 3–5). Arrhenius analysis of this remodeling shows that the salt-induced reduction in VLDL stability is an enthalpic effect (Figures 6 and 7). This is consistent with the ionic screening mechanism indicated by the dependence of the apparent melting temperature *T_m_* on the salt concentration (Figure 5(c)); in fact, ionic screening of electrostatic interactions within the lipoprotein is expected and observed to reduce the enthalpy of lipoprotein remodeling. It is also consistent with the negligible effect of salt-induced changes in the water activity on VLDL stability evident from the absence of any effect of 10% glucose on VLDL stability. We conclude that electrostatic interactions help stabilize the VLDL assembly; hence, screening of these interactions by the salt ions reduces VLDL stability. 

What groups form favorable electrostatic interactions in VLDL? Since most lipoprotein charges are located on the protein, the dominant effect is probably due to Coulombic protein-protein interactions, such as the putative salt bridge networks in class-A apolipoprotein *α*-helices [22]. Even though surface salt bridges are not necessarily stabilizing, their optimized networks can confer high structural stability to proteins [36–38] and, potentially, to lipoproteins [23, 24]. In addition, interactions between phospholipid head groups and/or free fatty acids with the basic residues, which in class-A helices are located at the polar-apolar interface in close proximity to lipid [21, 22], may also be important. The role of class-A *α*-helices is further supported by the comparison of the ionic strength effects on the structural stability of various classes of human lipoproteins. VLDL and HDL show similar effects of salt and, hence, are stabilized by favorable electrostatic interactions [24]. In contrast, LDL stability shows only a small change upon increasing salt concentration from 5 to 500 mM (Figure S2), suggesting that the net effect of the electrostatic interactions on LDL stability is much smaller than that in VLDL or HDL. As HDL contain only exchangeable apolipoproteins, LDL only the nonexchangeable apoB, and VLDL both exchangeable and nonexchangeable apolipoproteins, we speculate that the favorable electrostatic interactions observed in VLDL and HDL result, in part, from the abundance of the exchangeable apolipoproteins on the surface of these particles. High content of class-A *α*-helices in these proteins facilitates formation of extensive inter- and intrahelical salt bridge networks on the surface of HDL and VLDL. Conformational flexibility that is characteristic of the exchangeable apolipoproteins (reviewed in [39]) may help optimize the geometry of such salt bridge networks, while the low dielectric at the lipid-water interface is expected to amplify their effect on the lipoprotein stability.

### 4.2. Reduction in Stability at Mildly Acidic pH Is a Distinct Property of VLDL

VLDL is the only human lipoprotein that is destabilized upon lowering the pH from 8 to 6 (Figure 4). In contrast, the stability of human HDL and LDL does not significantly change in this pH range [24, 33]. To our knowledge, the only lipoproteins reported to have pH-dependent stability are binary complexes of human apoC-I, the smallest human apolipoprotein of 6 kD, with dipalmitoyl (16 : 0, 16 : 0) or distearoyl (18 : 0, 18 : 0) phosphatidylcholine [40]. Such reconstituted lipoproteins contain approximately 15–20 protein molecules per particle. Similar to VLDL, the stability of these apoC-I-containing complexes decreases upon reduction in pH from 8 to 6, with the midpoint near pH 7.2 [40]. In contrast, similar complexes containing apoA-I, the major HDL protein of 28 kD, show no pH effects on their stability [40]. We proposed that these effects result, in part, from the titration of the N-terminal *α*-amino group. Numerous copies of such amino groups are present on particles containing apoC-I [14–19], as opposed to 2–4 copies of apoA-I per particle; hence, particles containing apoC-I but not apoA-I show large changes in their stability upon titration of these amino groups at near-neutral pH [40]. Since each VLDL particle contains multiple copies of the exchangeable proteins on their surface (>20), we speculate that protonation of the N-terminal *α*-amino groups in these proteins contributes to the reduced VLDL stability at mildly acidic pH. Compared to VLDL, HDL and LDL contain some of the same proteins and lipids, yet they have only few protein molecules per particle (2–6) and, hence, only few *α*-amino groups. This may explain why only VLDL, but not HDL or LDL, show significant pH-dependent changes in their stability at near-neutral pH. Titration of multiple His in apoB (that adopts different conformation on different-size particles, such as LDL and VLDL) and apoE may also contribute to the observed pH effects on VLDL stability. Furthermore, compared to HDL and LDL, VLDL have higher content of free fatty acids whose titration at near-neutral pH is also expected to contribute to the observed pH effect on VLDL stability.

### 4.3. Potential Physiologic Implications

Destabilization of VLDL upon transfer from mildly basic to mildly acidic conditions is particularly pronounced at pH < 6. In fact, at pH 5.7, VLDL disintegration was observed at ambient temperatures even in low-salt solutions (data not shown). This may have important physiologic implications, since such ionic conditions are encountered *in vivo* during VLDL remodeling and catabolism. One example is the degradation of VLDL and LDL via the whole-particle endocytosis, in which the lipoproteins are first transferred from plasma (pH 7.4) to early endosomes (pH = 5.5–6.0). In contrast to LDL that undergo lysosomal degradation, VLDL degradation starts in the low-salt mildly acidic endosomal environment and involves dissociation of the exchangeable apolipoproteins such as apoE together with some lipid; this process is essential for apoE recycling and biogenesis of an important apoE-containing HDL fraction [10–12]. The remaining apoB-containing particles undergo lysosomal degradation. Reduction in VLDL stability upon reduction in pH from 7.4 to 6 reported here is expected to facilitate their endosomal degradation, specifically, the dissociation of the apoE-containing fraction from the apoB-containing particle (Figure 1). 

Another context in which a reduction in pH below neutrality may promote VLDL disintegration is atherosclerotic plaques. According to the widely accepted “response to retention hypothesis” of atherosclerosis ([14–16] and references therein), retention of LDL and VLDL in the arterial wall leads to their fusion and coalescence into small lipid droplets which are digested by macrophages; this triggers a cascade of proatherogenic and proinflammatory responses and culminates in the formation of foam cells and early atherosclerotic plaques. Such plaques contain lipoprotein-derived lipid droplets similar to those formed upon the heat-induced VLDL rupture (Figure 3(c)) [17, 18]. The near-neutral extracellular pH in early plaques becomes progressively acidic in more advanced plaques ([30] and references therein). We propose that such acidic environment destabilizes VLDL, enhancing their fusion and coalescence into large lipid droplets, such as the droplets found in the advanced atherosclerotic plaques. 

Furthermore, potential use of lipoproteins as carriers of lipophilic drugs depends critically on the structural stability of the carrier in plasma and in the target cells, particularly, its pH sensitivity [41, 42]. Our stability studies of plasma lipoproteins at pH 7.6 suggest that the lipoprotein stability tends to decrease with increasing particle size, from HDL to LDL to VLDL [20, 24, 33, 43]. Additional reduction in stability of larger particles such as VLDL at mildly acidic pH is one of the many factors to be considered when choosing a lipoprotein-based carrier for delivery of diagnostic or therapeutic agents to specific targets, such as the acidic microenvironment of solid tumors [44].

## Supplementary Material

The supplementary material includes the melting data illustrating the destabilizing effects of TRIS on VLDL assembly and the effect of salt on LDL stability.

## Abbreviations

VLDL: Very low-density lipoprotein
LDL: Low-density lipoprotein
HDL: High-density lipoprotein
Apo: Apolipoprotein
TG: Triacylglycerol
CD: Circular dichroism
T-jump: Temperature-jump
EM: Electron microscopy.
