# Supplementary material for: Human Plasma Very Low-Density Lipoproteins Are Stabilized by Electrostatic Interactions and Destabilized by Acidic pH
Source: J Lipids. 2011 May 15;2011:493720. doi: 10.1155/2011/493720 (PMC3136112; doi:10.1155/2011/493720)
Supplement: Supplementary file 1 — The supplementary material includes the melting data illustrating the destabilizing effects of TRIS on VLDL assembly and the effect of salt on LDL stability. [file 493720.f1.pdf]

Human Plasma Very Low-Density Lipoproteins are Stabilized by Electrostatic Interactions and Destabilized by Acidic pH

Madhumita Guha & Olga Gursky

SUPPLEMENTAL DATA

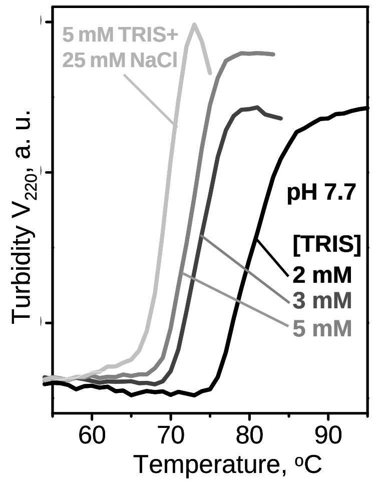

Figure S1. Destabilizing effects of TRIS on VLDL assembly. VLDL samples containing 0.1 mg/mL protein at pH 7.7 in TRIS buffer (2, 3 or 5 mM as indicated) with or without 25 mM NaCl were heated at a constant rate of 11 °C/h. Thermal denaturation of VLDL, which involves particle fusion and coalescence into lipid droplets, was monitored by turbidity at 220 nm. Increasing TRIS concentration from 2-5 mM shifts VLDL denaturation to lower temperatures by about 10 °C, indicating a large destabilizing effect of TRIS.

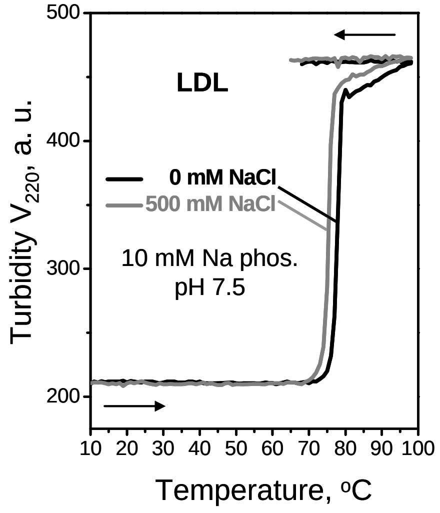

Figure S2. Salt has relatively little effect on LDL stability. Human plasma LDL samples (3.5 mg/mL protein in 10 mM Na phosphate buffer, pH 7.5) containing 0 or 500 mM NaCl were heated and cooled at a constant rate of 11 °C/h. Thermal denaturation, which involves LDL fusion and coalescence into lipid droplets, was monitored by turbidity. The results showed a small ~3 °C reduction in the apparent transition temperature T_m upon increasing NaCl concentration from 0-500 mM. This and other similar experiments suggested little changes in LDL stability upon increasing Na salt concentration.
